# A large-size and polarization-independent two dimensional grating fabricated by scanned reactive-ion-beam etching

**DOI:** 10.1515/nanoph-2022-0371

**Published:** 2022-09-29

**Authors:** Wei Zhang, Wenhao Li, Tong Zhang, Zhongming Zheng, Zhendong Chi, Yanxiu Jiang, Na Wu

**Affiliations:** Changchun Institute of Optics, Fine Mechanics and Physics, Chinese Academy of Sciences, No. 3888 Dong Nanhu Road, Changchun, Jilin, 130033, China

**Keywords:** large size grating, polarization independent, scanned reactive-ion-beam etching, two-dimensional grating

## Abstract

Scanned reactive-ion-beam etching method was proposed to transfer two-dimensional mask patterns into quartz substrate, which would produce a larger-size and polarization-independent two-dimensional grating. This method was realized by moving grating substrate in a unidimensional scanning manner and adjusting ion beam density in the vertical scanning direction. Graphite plates between the ion beam source and the substrate were used to correct the beam density. The original Gaussian ion beam density was changed to a uniform distribution to establish a knife-edge shape around the vertical scanning direction. Therefore, a large-area pattern with consistent depth and duty cycle would be engraved into a quartz substrate. A two-dimensional, 1200 groves/mm grating with an 85-mm × 85-mm area was fabricated under scanned reactive-ion-beam etching method and exhibited a 0.197*λ* (*λ* = 632.8 nm) diffraction wave front. At 780 nm, the efficiency nonuniformity was less than 9%, and the average diffraction efficiencies of transverse-magnetic and transverse-electric polarized light were 57.2 and 58.0%, respectively. The large-size two-dimensional grating with uniform diffraction efficiency and polarization independence enabled grating displacement measurement with high resolution, long measurement range, multiple degrees of freedom, and potential miniaturization.

## 1 Introduction

Ultra-precision displacement measurement technology is a foundation of modern manufacturing. It requires equipment with long measurement range and multiple degrees of freedom []. Grating measurement technology based on diffracted-light interference has become of great interest in the area of ultra-precision displacement measurement. Using a grating as the core component enables a compact structure and a short optical path, which is less affected by the external environment and results in highly reliable measurement. Interferometric displacement measurement via diffraction grating have been the preferred technique for nanoscale motion resolution, with high precision, long measurement range, multiple degrees of freedom, and miniaturization [].

The resolution and measurement range of a grating displacement measurement system is affected by the groove density and grating size. A grating with a high groove density exhibits high-resolution and high-precision displacement measurement [14–16]. However, it is difficult to balance high groove density and large size, with the result that high-resolution and high-precision system have short measurement range [17]. On the other hand, fabrication of multi-dimensional grating is also very challenging. Hence, increasing measurement degree of freedom would lead to more complex structure, lower accuracy, and less reliability, which are not conducive to miniaturization [18]. For example, by using a dual one-dimensional grating as a reference in a Michelson interferometer structure, or by using a Littrow-incidence one-dimensional grating for homodyne measurement, a displacement measurement system could expand the degree of freedom. However, the complexity creates low reliability and increased interference, which is not suitable for integrated installation [19–22].

Hence, two conflicts arise for grating displacement measurement system. One is the conflict between long measurement range and high resolution, and the other is between multiple degrees of freedom and miniaturization. Improving the dimension and size of a high-groove-density grating is one way to solve these problems. Two-dimensional grating with large size, high groove density, and high precision could enable high-resolution displacement measurement with multiple degrees of freedom, long-range displacement, and miniaturized structure.

So far, it is still very challenging to fabricate two-dimensional grating with area larger than 50 mm × 50 mm. Two-dimensional grating with 16% diffraction efficiency at normal incidence was fabricated previously [23–25]. For grating displacement measurement, low diffraction efficiency results in low signal-to-noise ratio and poor precision. Furthermore, with the increased groove density in a two-dimensional grating, incident light polarization would significantly affect the contrast of signal in a displacement measurement system [24]. Therefore, complex structure for two-dimensional grating has been proposed for high diffraction efficiency without polarization dependence. Chen et al. proposed a sandwiched structure with a 200-nm-thick Au layer placed between a quartz substrate and a 280-nm deep HfO_2_ grating layer. The high-refractive-index HfO_2_ grating with a 50-mm × 50-mm area was fabricated with holographic exposure and reactive-ion etching. Diffraction efficiencies for transverse-magnetic (TM) and transverse-electric (TE) polarized light were 74.8 and 68.2%, respectively, over the wavelength range 770–790 nm, which ensured polarization-independent, high diffraction efficiency. However, nonuniformity in the diffraction efficiency at full aperture has not been reported [23]. To improve the diffraction efficiency, Zhou et al. proposed a four-layer structure consisting of a quartz substrate, a metal layer, a low-refractive-index dielectric layer, and high-refractive-index dielectric grating layer in sequence. The metal layer was a 200-nm-thick Ag film, and the low-refractive-index dielectric layer was a 520-nm-thick SiO_2_ film. The groove depth of the Ta_2_O_5_ grating was 556 nm, which was created with laser direct writing combined with reactive-ion etching. At 780 nm, the diffraction efficiency of TM- and TE-polarized light was 98.31 and 98.05%, respectively. High diffraction efficiency with polarization independence was thus realized, but the grating size and the diffraction-efficiency uniformity at full aperture was not reported [24]. As mentioned above, to improve the diffraction efficiency, their grating structure would become very complex. Moreover, the grating grooves were not directly engraved into the quartz substrate, which would reduce the reliability of a two-dimensional grating because of film durability. In 2021, Zhou et al. simplified the two-dimensional grating structure by directly etching the grooves on the quartz substrate via holographic exposure and reactive-ion etching, followed by an Au film coating on the grating [25]. The grating area was only 25 mm × 25 mm and had an 18% diffraction efficiency at 780 nm under normal incidence.

At present, for large-size (larger than 50 mm × 50 mm) two-dimensional grating engraved into quartz substrate, groove consistency in the whole aperture is a key problem to be solved. At the same time, when the size of grating increases, it is necessary to use thick quartz substrate (generally at least greater than 15 mm thick) to ensure high quality of diffraction wavefront. However, nonconductive property of thick quartz substrate leads that inconsistent plasma distribution for reactive ion etching method which is the pattern transfer technique in the microelectronics semiconductor industry. Thus, reactive ion etching method is not suitable for the grating fabrication of thick quartz substrate. Reactive ion beam etching method with ion beam excited by ion source is independent of the grating substrate thickness, thus has been widely used for pattern transfer of thick quartz grating. However, the area of ion beam is determined by the size of ion beam source and the distribution of ion beam is Gaussian, so the area of grating groove consistency is still restricted. Although inhomogeneity of grating groove caused by Gaussian ion beam distribution could be alleviated by using a correction mask to modify ion beam profile [26, 27], the limit of grating size by the diameter of ion source has not been broken. Therefore, it is necessary and urgent to develop a processing method to break the limitation of grating size by ion beam source aperture and realize the two-dimensional grating with large size, uniform groove shape and polarization independent.

Here, scanned reactive-ion-beam etching method was proposed to engrave a large two-dimensional grating in a quartz substrate with uniform, polarization-independent diffraction efficiency. The groove shape was designed for polarization independence. The two-dimensional pattern was recorded in the photoresist mask with two orthogonal holographic exposures. Scanned reactive-ion-beam etching method transferred the mask pattern into the quartz substrate. During the etching process, grating substrate is moved with respect to ion beam source in one-dimensional scanning mode. In the vertical scanning direction, the ion-beam density with Gaussian distribution was made more uniform with a graphite baffle placed between the ion beam source and the grating substrate, such that the ion-beam density possessed a knife-edge distribution incident on the substrate. The two-dimensional grating with an 85-mm × 85-mm full-aperture area had diffraction wave front of 0.197*λ* (*λ* = 632.8 nm), and less than 9% nonuniformity in the diffraction efficiency. The average diffraction efficiency of TM and TE-polarized light was 57.2 and 58.0%, respectively; thus, the polarization imbalance was 0.69%. The two-dimensional grating is expected to be used in the field of displacement measurement and solve the conflict between multiple degrees of freedom and miniaturization.

## 2 Grating design

**Figure 1:**
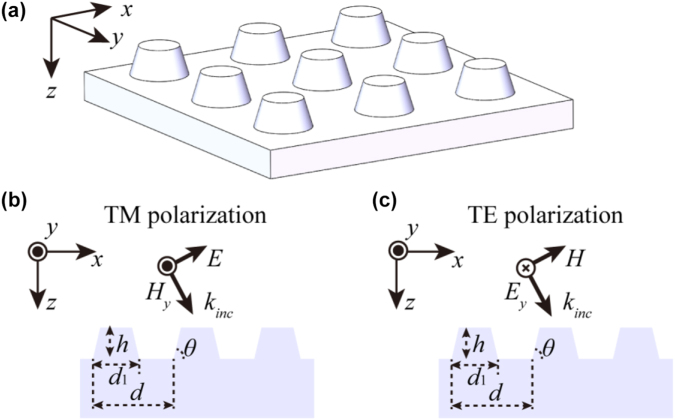
Schematic of two-dimensional grating structure. (a) 3D structure of two-dimensional grating, (b) and (c) represent the grating with transverse-magnetic (TM) and transverse-electric (TE) polarized incident light in *xz* plane.

A schematic of the two-dimensional grating structure is shown in Figure 1. The groove pattern had an orthogonal distribution with a 1200-grooves/mm density (period *d* = 833 nm). 780-nm light incident in a Littrow manner had an angle of 27.9°. The substrate was quartz and Al was used as the grating reflection film. According to the previous experience, the actual fabrication condition leads to a trapezoidal shape ridge for one-dimensional grating. Therefore, a circular truncated cone with an 80° sidewall inclination was used as the two-dimensional grating groove shape. The depth *h* and bottom width *d*_1_ of a grating groove are shown in Figure 1, and the duty cycle of the groove bottom was *f* = *d*_1_/*d*. A finite-difference time-domain method was used to optimize the parameters of two-dimensional grating structure. The calculation model had Bloch periodic boundary conditions around the structure, and the upper and lower layers were set as perfectly matched layer. At the wavelength of 780 nm, the refractive indices of the quartz and the Al coating [28] were 1.52 and 2.63 + 8.6*i*, respectively.

**Figure 2:**
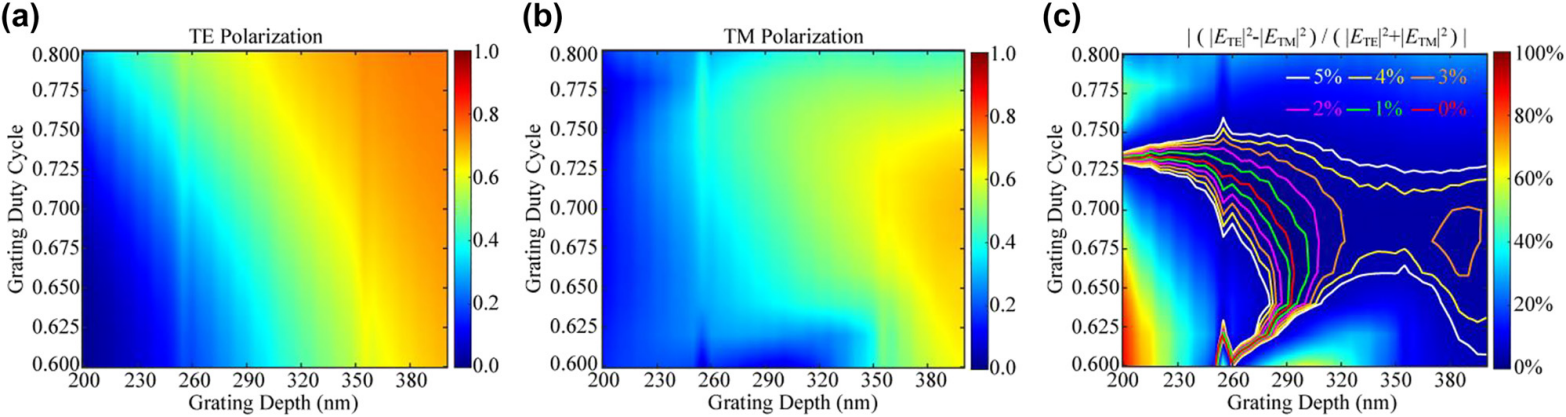
Diffraction efficiency of (−1, 0)-order diffracted light for the two-dimensional grating versus groove depth *h* and bottom duty cycle *f* under (a) transverse-electric (TE) and (b) transverse-magnetic (TM) polarization. (c) Evaluated imbalance of diffraction efficiency between TE and TM. Contour lines with different colours represent the levels of polarization-dependent diffraction efficiency.

Figure 2(a) and (b) show the simulation results of the diffraction efficiency of (−1, 0)-order diffracted light versus groove depth *h* and groove-bottom duty cycle *f* for TE and TM polarization. The grating groove depth had a significant effect on the diffraction efficiency, regardless of the polarization, which was consistent with theory. Also, the duty cycle had a more significant effect on TM-polarized diffraction efficiency than on that for TE polarization. The equation | (|*E*_TE_|^2^ − |*E*_TM_|^2^)/(|*E*_TE_|^2^ + |*E*_TM_|^2^) | was defined to evaluate the imbalance of TE and TM diffraction efficiency, where |*E*_TE_|^2^ and |*E*_TM_|^2^ were the electric-field intensities of (−1, 0)-order diffracted light for TE and TM polarization, respectively. The imbalance was calculated in Figure 2(c), where the red solid line indicated equal diffraction efficiency for TE and TM polarization. Along the red solid line, the diffraction efficiency reached a maximum of 42.3% at a 285-nm groove depth and a 0.69 duty cycle. From Figure 2, the diffraction efficiency under TE and TM polarization could again be equal with an increased grating groove depth, but grating with deep grooves would significantly increase the fabrication difficulty. Various imbalance evaluations are exhibited by different-colored contour lines in Figure 2(c). Around a 290-nm grating depth in the distribution of contour lines, the polarization-independent diffraction efficiency was less affected by the variation in depth and duty cycle. In general, control of the duty cycle is more difficult than that of groove depth in grating fabrication. Thus, grating parameters with an optimized region around a 0.69 duty cycle and a 290-nm depth were used for the consideration of duty cycle with greater process tolerance, where the diffraction efficiency would approach 55%.

## 3 Grating fabrication

**Figure 3:**
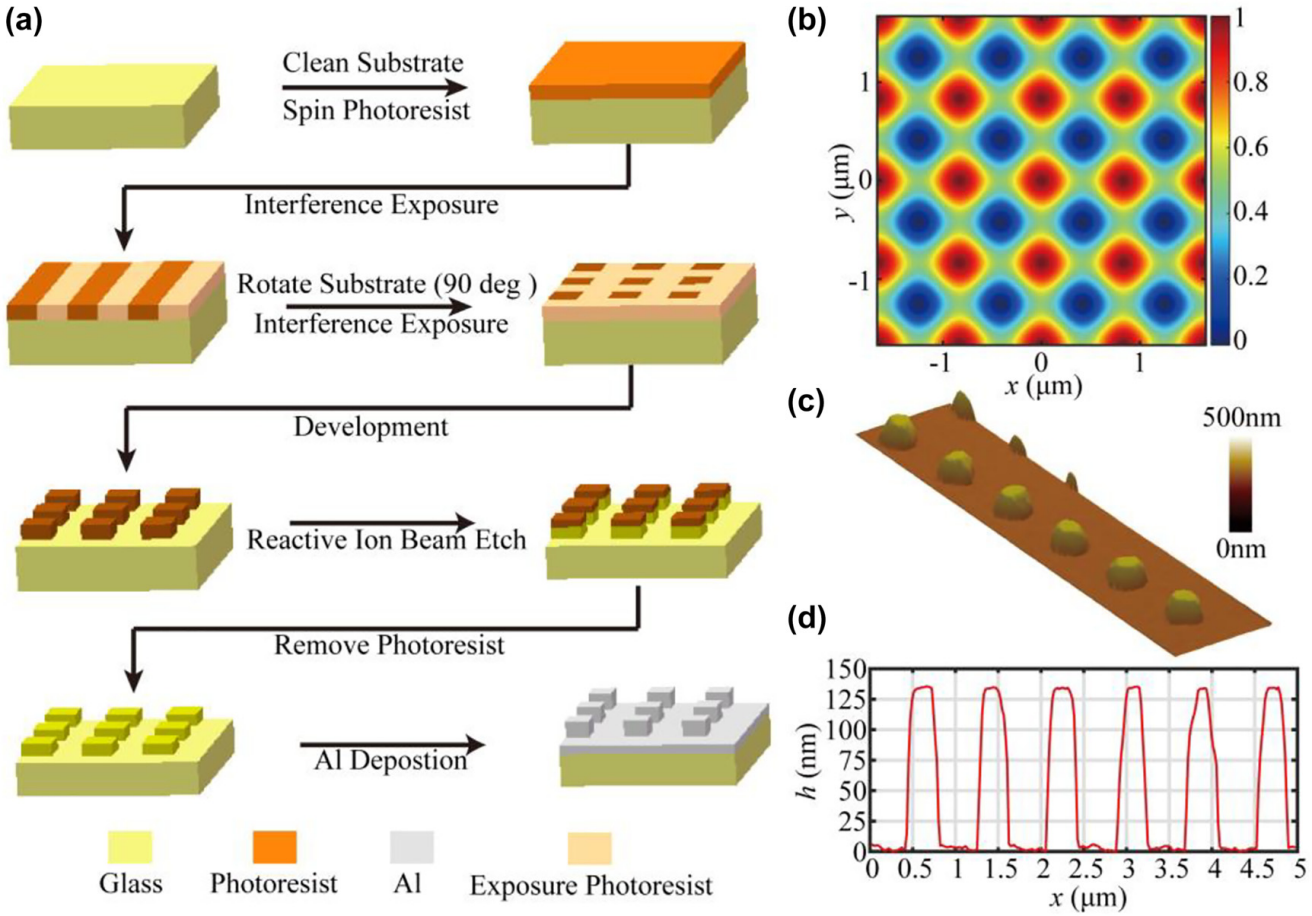
Fabrication of two-dimensional grating. (a) Process flow in two-dimensional grating fabrication; (b) normalized electrical intensity distribution in the superposition of field intensities for the two orthogonal interference exposures. (c) Atomic microscopy image and (d) cross-section graph of the photoresist mask.

Figure 3(a) shows the flow chart for the two-dimensional grating fabrication. The quartz substrate had an area of 85 mm × 85 mm and a thickness of 16 mm. It had a wave front less than 0.1*λ* (*λ* = 632.8 nm) and was ultrasonically cleaned with acetone and deionized water. The 120-nm-thick S1805 photoresist was spun onto the substrate, and a large-aperture amplitude-division exposure system was built with a 441.5-nm laser source and 250-mm-diameter aspherical collimating lens. The grating groove density was determined by the angle between two collimating beams in the exposure system, which was adjusted with a Moiré alignment algorithm [29, 30]. The exposure system formed a one-dimensional interference fringe with a 200-mm-diameter consistent contrast area. Subsequently, the quartz substrate and photoresist were exposed to the interference fringe field to record the two-dimensional pattern. The pattern could be formed by rotating the substrate for two orthogonal exposures. Each exposure had duration of 5 min. Figure 3(b) simulates the electric-field intensity distribution for the superimposition of two orthogonal interference exposure fields, which shows that the grating fringes were two-dimensional orthogonal cylindrical grooves after the exposures. The exposed photoresist was developed for 50 s in a solution with 3.6 g of NaOH in 1 L of deionized water. After flushing with deionized water and drying under nitrogen, the photoresist mask was obtained. The grating groove of the mask was imaged with an atomic force microscope, as shown in Figure 3(c). The groove depth and duty-cycle ratio of the groove bottom were 131.2 nm and 0.52, respectively (Figure 3(d)).

**Figure 4:**
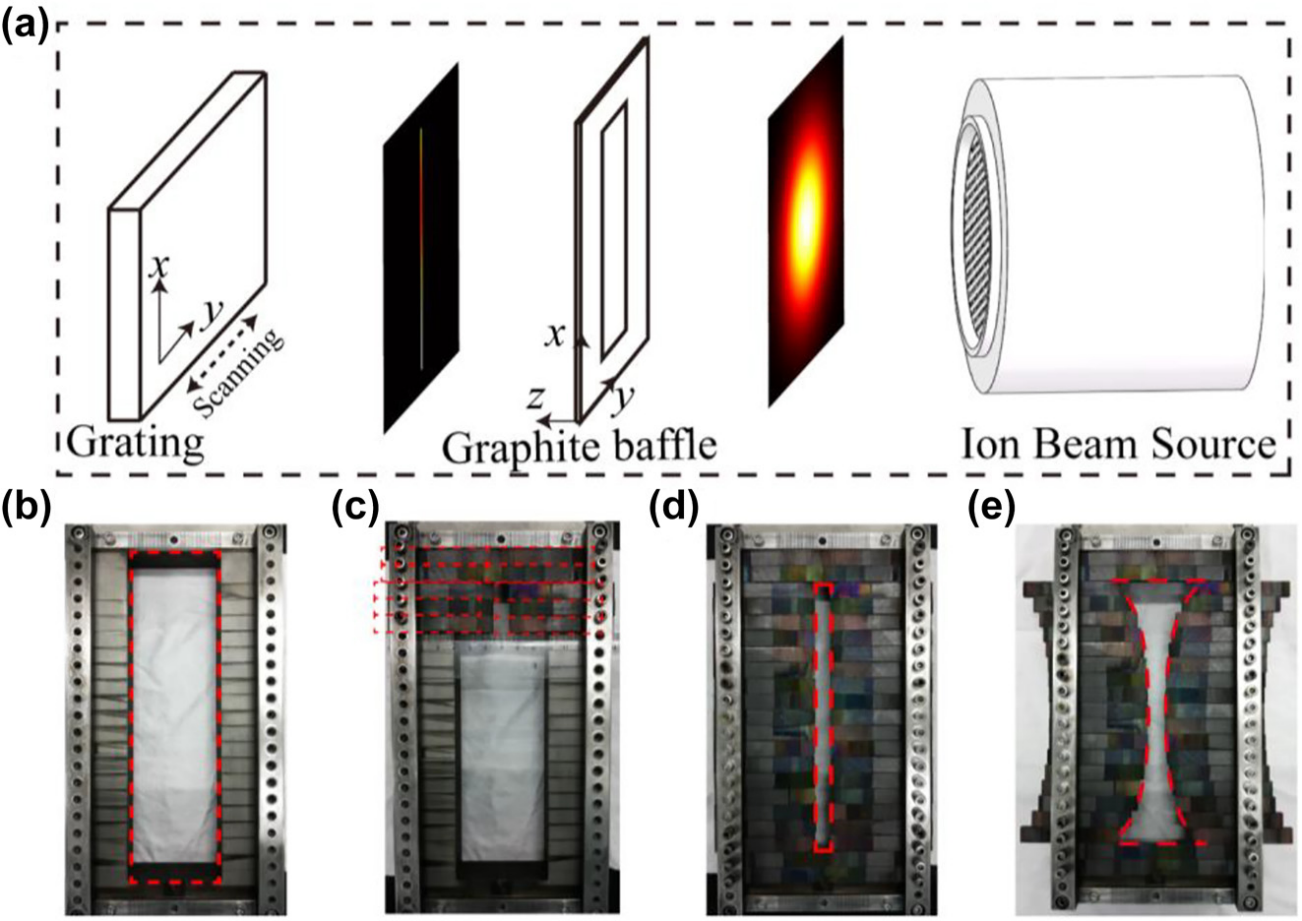
Scanned reactive-ion-beam etching method. (a) Schematic of scanned reactive-ion-beam etching for large-area grating. (b) Photograph of the graphite baffle holder. Red-dotted box indicates the opening area of the holder structure. (c) Adjusting the graphite plates, as shown by red-dotted box to change the shape of the graphite baffle. (d) Rectangular and (e) hyperbolic graphite baffles.

The photoresist mask was then exposed to the reactive-ion beam that etches the mask pattern into the quartz substrate. A schematic of the scanned etching for large-area grating is shown in Figure 4(a). The ion source had a beam diameter of 160 mm. To improve the etching selectivity ratio of the substrate and photoresist, a mixture of Ar and CHF_3_ gases was used for the ion source. So, the ion beam had Ar^+^ and CHF_2_^+^. Generally, the Gaussian distribution of the ion beam density resulted in an inconsistent groove shape. A uniform ion beam was the key for making large-area patterns. An external and shape-controlled graphite baffle between the ion source and the substrate was therefore used to adjust the ion-beam density distribution. By optimizing the opening shape of the graphite baffle, a uniform distribution was achieved, with a “knife-edge” shape along the *x*-axis direction. Finally, the modified ion beam impinged on the photoresist mask while scanning along the *y*-axis direction.

The external graphite baffle was installed in a holder with a 210-mm × 60-mm opening, as shown in Figure 4(b). The baffle shape was changed by adjusting the positions of multiple graphite plates. The red-dotted box in Figure 4(c) shows a 65-mm × 10-mm graphite plate, which was fixed to the holder structure. Rectangular and hyperbolic openings are shown in Figure 4(d) and (e), respectively.

**Figure 5:**
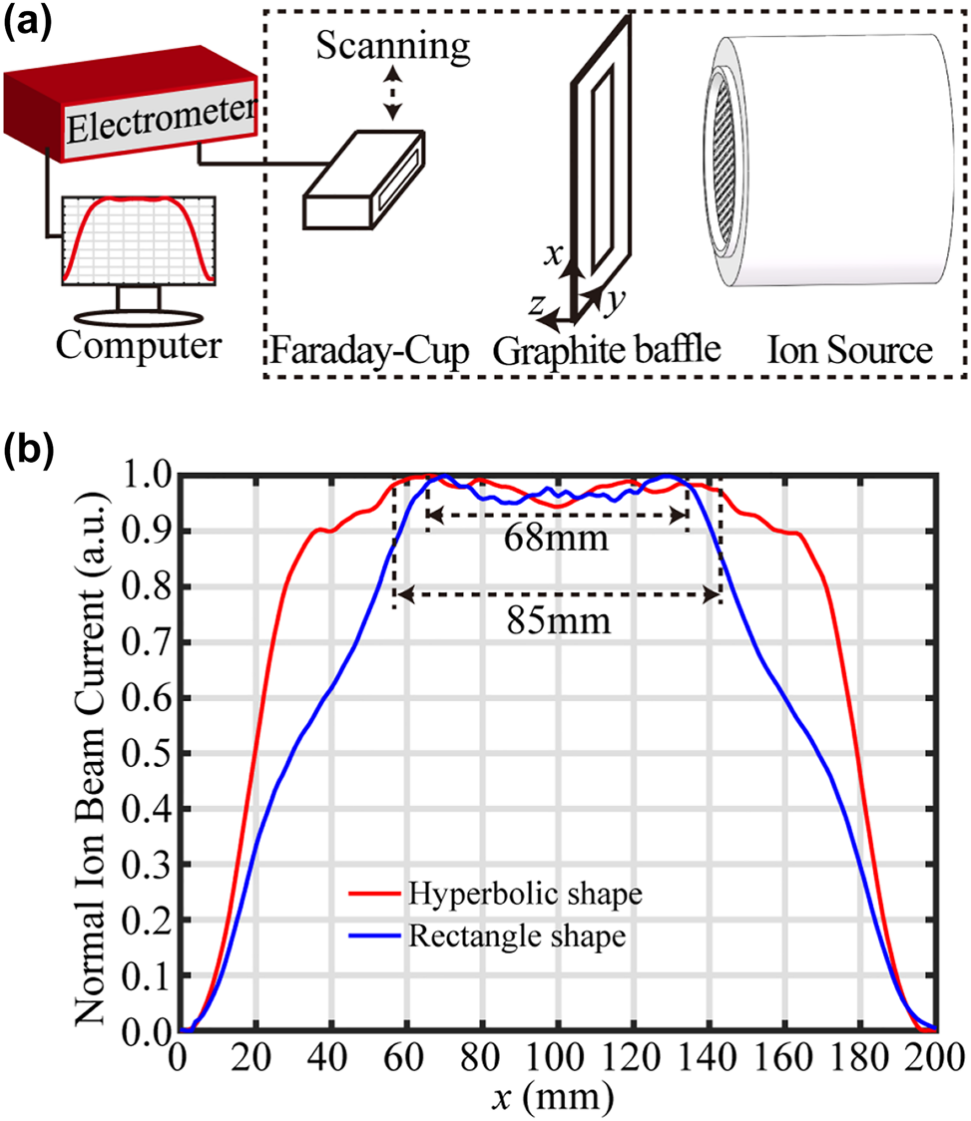
Density distribution of the ion-beam. (a) Method to test the ion-beam density distribution. The region enclosed by the dashed line was inside a vacuum chamber. (b) Distribution curves of ion-beam density for graphite baffles with the rectangular and hyperbolic shape opening.

To evaluate the ion-beam density distribution, a rectangular Faraday cup was used to collect the ion current, as exhibited in Figure 5(a). The probe head of the Faraday cup had an 85-mm-long slit along the *y*-axis and a 10-mm width along the *x*-axis. Inside the probe head was an ion collector with a U-shaped graphite strip, and the Faraday cup was connected to a Keithley 6517 electrometer, which yielded a reference current for the ion beam density. The ion-beam density distribution was obtained by scanning the Faraday cup along the *x*-axis direction. The distribution of ion beam acting on the grating substrate was measured *in situ* under the same condition as etching. The ion-beam current distribution for the graphite baffle with rectangular opening is depicted by the blue curve in Figure 5(b), and its uniform area was approximately 68 mm. When the opening of graphite baffle was changed to the hyperbolic aperture, the resulting ion beam distribution was as shown by the red curve in Figure 5(b), with a uniform area of approximately 85 mm. Hence, the external graphite baffle improved the uniformity of the ion beam density.

## 4 Results

**Figure 6:**
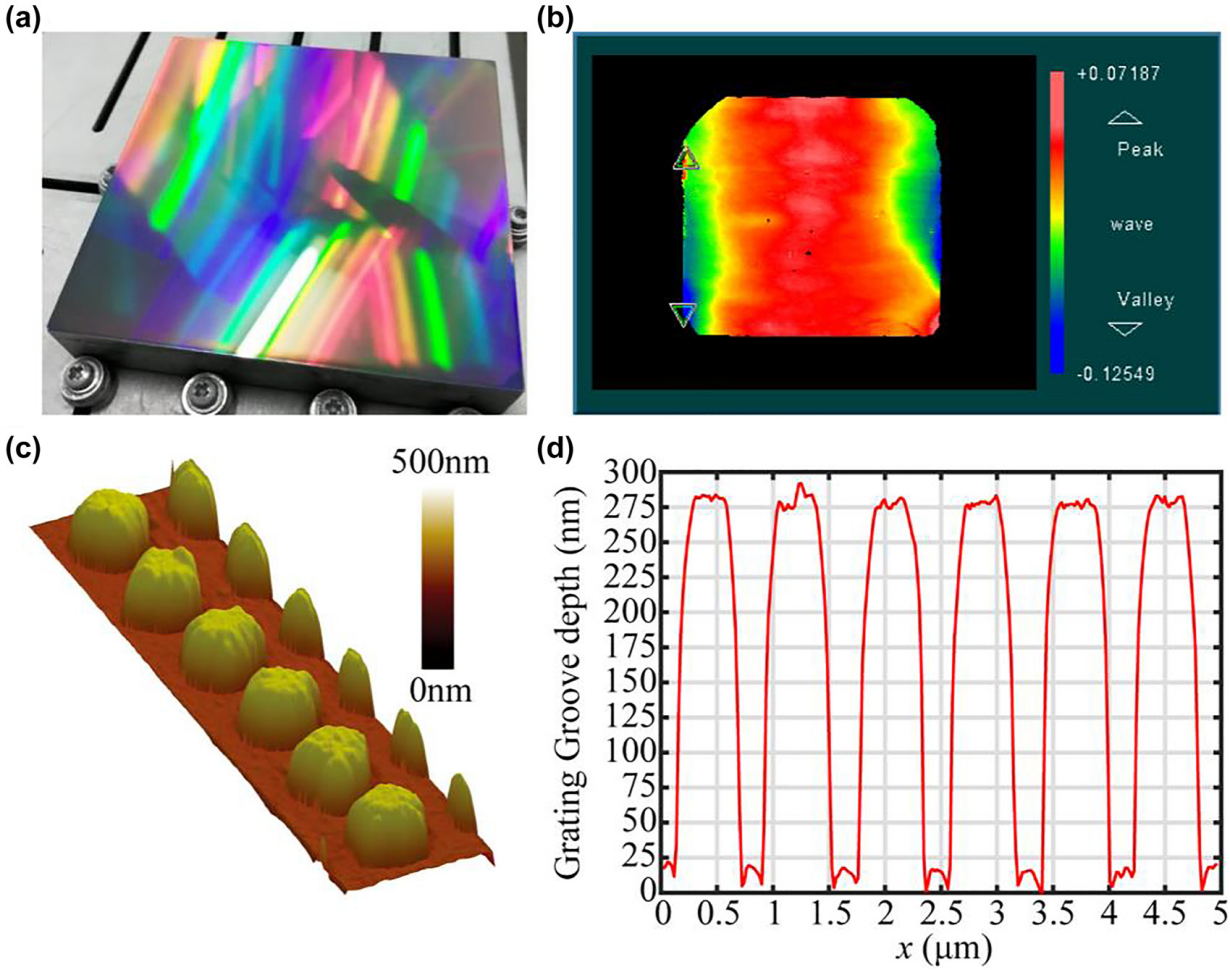
The frabricated two-dimensional grating. (a) Photograph of the two-dimensional grating made via scanned reactive-ion-beam etching (b) Diffraction wave front measured with a Zygo interferometer. (c) Atomic force microscope image and (d) cross-sectional view of the two-dimensional grating.

An 85-mm × 85-mm two-dimensional grating is shown in Figure 6(a); it had a 150-nm-thick sputter-coated Al film on top. A diffraction wave front was 0.197*λ* (*λ* = 632.8 nm) in the full 85-mm × 85-mm area, as measured with a Zygo interferometer (Figure 6(b)). An atomic force microscope image of the grating is shown in Figure 6(c), and the corresponding cross section is shown in Figure 6(d). The groove shape of the grating was a circular truncated cone with a 275-nm depth, a duty-cycle ratio of 0.71, and an 82° sidewall inclination angle.

**Figure 7:**
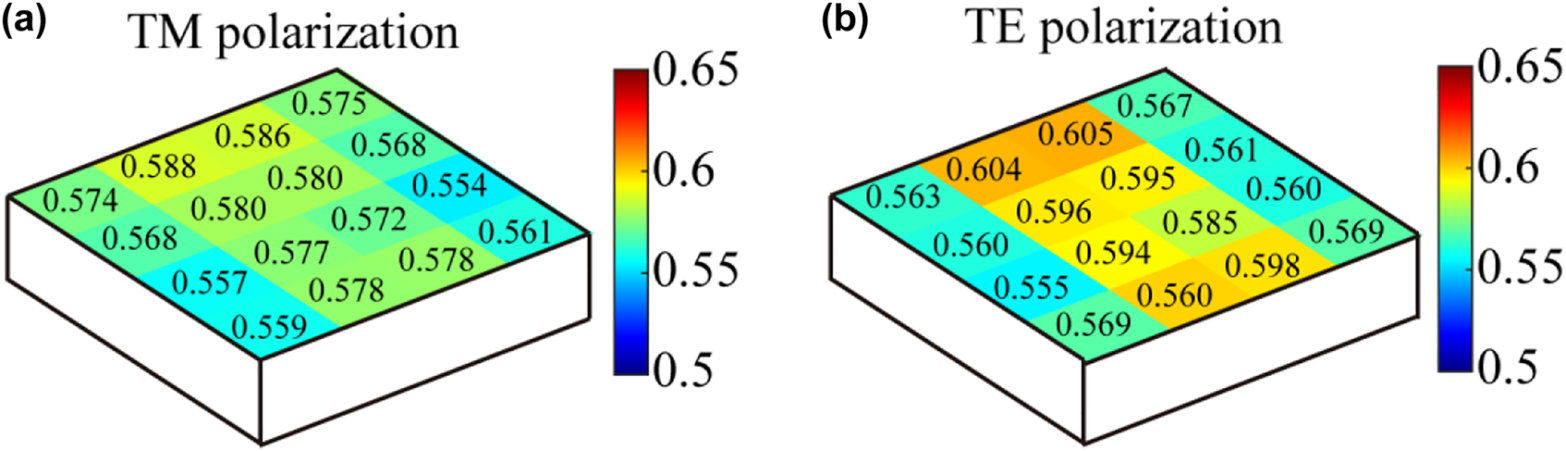
Experimental results of the (−1, 0)-order diffracted-light reflection efficiency under (a) transverse-magnetic and (b) transverse-electric polarizations for the two-dimensional grating. Sixteen points were distributed on the full-aperture area in a 4 × 4 array.

The reflection efficiency of the (−1, 0)-order diffracted light was measured with a 780-nm laser with sixteen points in a 4 × 4 array on the full-aperture two-dimensional grating. Different polarization states were observed by rotating the laser 90°. The point-by-point results of the diffraction efficiency with the movement of the grating are shown in Figure 7(a) and (b) for different polarization states. The diffraction efficiency at the different positions on the grating was represented by *η*_*i*_. The average efficiency, nonuniformity of the efficiency, and polarization imbalance were, respectively, defined as:

$$Average\;\;Di\mathrm{ff}raction\;\;E\mathrm{ff}ciency=\frac{1}{n}\overset{n}{\underset{i=1}{\sum }}\eta_{i}$$

$$\begin{array}{ll} & Nonuniformity\;\;of\;\;Di\mathrm{ff}raction\;\;E\mathrm{ffi}ciency\\ & \;=\frac{\max\left(\eta_{1},\eta_{2},\cdots ,\eta_{n}\right)-\min\left(\eta_{1},\eta_{2},\cdots ,\eta_{n}\right)}{\frac{1}{n}\overset{n}{\underset{i=1}{\sum }}\eta_{i}} \end{array}$$

$$Polarization\;\;Imbalance=\left|\frac{\eta_{TE}-\eta_{TM}}{\eta_{TE}+\eta_{TM}}\right|$$

From the test results in Figure 7, average diffraction efficiency, nonuniformity of diffraction efficiency, and polarization imbalance of the two-dimensional grating were calculated. The average diffraction efficiencies for the TE and TM polarizations were 57.2 and 58%, respectively. Within the 85-mm × 85-mm area, the nonuniformity of diffraction efficiency under TE and TM polarization was less than 9%, and the polarization imbalance was 0.69%.

## 5 Conclusions

Scanned reactive-ion-beam etching method was demonstrated to fabricate a large-size two-dimensional grating with a uniform diffraction efficiency and polarization-independent behavior. The two-dimensional grating had an area of 85-mm × 85-mm with a full-aperture diffraction wave front of 0.197*λ* (*λ* = 632.8 nm), and the nonuniformity of diffraction efficiency less than 9%. The average diffraction efficiency of TM and TE-polarized light was 57.2 and 58.0%, respectively, with a polarization imbalance of 0.69%. The grating developed by this method presented the expanded dimension and the enlarged size, which is potential for grating displacement measurement with increased degrees of freedom, an elongated measurement range, and a reduced system complexity.
